# Association of mental health in childhood, adolescence and young adulthood with cardiovascular risk factors and carotid remodeling below age 30 - results from the KiGGS cohort study

**DOI:** 10.1007/s10654-024-01189-3

**Published:** 2025-01-03

**Authors:** Julia Charlotte Büschges, Ann-Kristin Beyer, Arno Schmidt-Trucksäss, Klaus Berger, Hannelore Neuhauser

**Affiliations:** 1 Department of Epidemiology and Health Monitoring, Robert Koch Institute, Gerichtstr. 27, D-13347 Berlin, Germany; 2 DZHK (German Centre for Cardiovascular Research), Partner Site Berlin, Berlin, Germany; 3 Charité – Universitätsmedizin Berlin, Corporate Member of Freie Universität Berlin and Humboldt-Universität zu Berlin, Berlin, Germany; 4 Department of Sport, Exercise and Health, Division Sports and Exercise Medicine, University of Basel, Basel, Switzerland; 5 Department of Clinical Research, University Hospital Basel, University of Basel, Basel, Switzerland; 6 Institute of Epidemiology and Social Medicine, University of Münster, Munster, Germany

**Keywords:** Mental health, Cardiovascular risk, Adolescence, Carotid intima-media thickness, Carotid stiffness, Strengths and difficulties questionnaire

## Abstract

An association of mental health and in particular depression with cardiovascular disease has been shown in adults and to a lesser extent in the young. Recently improved measurement methods of carotid-intima media thickness (CIMT) and carotid stiffness (CS) allow more differentiated analyses of this link. We examined 4,361 participants of the nationwide KiGGS cohort aged 3–17 years at baseline and 14–28 years at follow-up. Using linear and logistic regressions, we analyzed cross-sectional and longitudinal associations of mental health with systolic blood pressure (SBP), body mass index (BMI) and total cholesterol (TC) as well as CIMT and CS from high-resolution carotid sonography at follow-up. Mental health in children was measured with the Strength and Difficulties Questionnaire (SDQ) and in adults with the Mental Health Inventory (MHI-5) and the Patient Health Questionnaire (PHQ-9). Childhood SDQ scores were associated longitudinally with SBP, BMI and TC (-0.03≤ ß≥ 0.02) but not with CIMT or CS one decade later. Similarly, SDQ at follow-up was associated cross-sectionally with SBP, BMI and TC, but not CIMT or CS. MHI-5 scores were not linked to any outcome. PHQ-9 scores in young adults were associated cross-sectionally with SBP and BMI (-0.26≤ ß≥ 0.01), but not with CIMT or CS. Our study shows that children, adolescents and young adults with impaired mental health also have an increased long-term cardiovascular risk through higher BMI and TC. However, in this sample with predominantly mild mental health impairments carotid remodeling was not evident.

**Supplementary Information:**

The online version contains supplementary material available at 10.1007/s10654-024-01189-3.

## Introduction

Cardiovascular disease (CVD) and depression are linked bidirectionally in adults through biological as well as behavioral pathways. Population-based cohort studies, for example the Whitehall II study [1], provide evidence that CVD, such as stroke and coronary artery disease, are associated with an elevated risk for later onset of depression in older adults. The vascular depression hypothesis proposes that atherosclerotic lesions may impair brain regions responsible for affect regulation, resulting in depressive symptoms [2, 3]. Findings from cohort studies on cerebrovascular risk factors [4] as well as carotid stiffness (CS) [5] and subsequent incident depression support this hypothesis. However, there is a large body of evidence also suggesting a reversed causal relationship. For instance, a pooled analysis of more than 500,000 participants from 22 cohorts led to the conclusion that the presence of depressive symptoms was associated with increased subsequent CVD incidence [6]. Depressive symptoms have also been linked to biomarkers of subclinical atherosclerosis, such as coronary artery calcification [7–9] and pulse-wave velocity (PWV) [10–12]. Similarly, several studies have proposed a link between depressive symptoms and vascular remodeling of the carotids, i.e. increased carotid intima-media thickness (CIMT) [13, 14] and CIMT progression [15, 16]. In addition, there is some evidence indicating that symptoms of anxiety [17] and anger [18] are linked to CIMT as well. This is in line with findings showing that not only depression, but also its frequent comorbid conditions of anxiety and chronic stress are linked to CVD [19, 20].

In young age groups, low mental health has been associated with unhealthy eating behavior, reduced physical activity, sleep disturbances and increased smoking [21–23], all of which are cardiovascular risk (CVR) factors. Prior studies linked the presence of such risk factors in childhood and adolescence to elevated CIMT in adulthood, more than one decade later [24–26].

However, such studies that examine mental health and subclinical cardiovascular outcomes in the young are scarce. Further, the range of subclinical outcomes is much smaller in the young than in middle-aged or older adults. It consists mainly of CIMT and, more rarely, PWV or retinal arteriole-to-venule ratio. While some studies suggested an association of emotional or more specifically depressive symptoms with increased CIMT in the young [27–29], other studies did not find evidence for an association [30–35].

Since atherosclerotic changes accumulate over a prolonged time period and preventive measures are most effective when targeted precisely and initiated early on, further investigations into this topic are highly relevant. The population-based KiGGS cohort provides the opportunity to examine the association of mental health in childhood, adolescence and young adulthood with CIMT and CS, as well as related CVR factors. Drawing on third-generation carotid sonography, the aim of this study is to address the following questions: (1) Is mental health related to CVR factors, i.e. systolic blood pressure (SBP), body-mass index (BMI) and total cholesterol (TC) in the young (age 14 to 28 years)? (2) Are symptoms of mental health problems in childhood (age 3 to 17 years) associated with higher CIMT and CS one decade later, thus age 14 to 28 years? (3) Is mental health in adolescence and young adulthood (age 14 to 28 years) associated cross-sectionally with CIMT and CS?

## Materials and methods

### Study population

From 2003 to 2006, the German Health Examination Survey for Children and Adolescents, abbreviated as KiGGS, was carried out as the first German nationwide health survey of a representative sample of children and adolescents up to age 17. Follow-up examinations of cohort participants took place 11 years later (2014–2017). KiGGS was approved by the federal Commissioner for Data Protection and Freedom of Information as well as two ethics committees (Charité Universitätsmedizin Berlin No. 101/2000; Medizinische Hochschule Hannover No. 2275 − 2014). At baseline and follow-up, both parents or guardians provided informed written consent following a personal dialogue and provision of written information. If a participant was aged 14 years or older and assessed as being capable of giving consent by the attending study physician, their informed written consent was also obtained. Independent of the participant’s age and the declarations of consent, interviews and examination were only carried out if assent of the participant was assured.

At follow-up, carotid sonography was successfully performed in 4,709 participants aged 14–28 years. Only participants with complete parent-reported SDQ at baseline were considered for further analyses (*n* = 4,636). 239 participants were excluded due to missing information on blood pressure (BP), BMI or TC at follow-up. Furthermore, 36 participants with neither parent-reported SDQ nor information on MHI-5 or PHQ-9 at follow-up were excluded. Thus, the sample includes 4,361 participants. Parameters of CS could be computed for 3,991 participants.

## Measures of mental health

At baseline and follow-up, parents of participants up to age 17 completed the parental version of the Strengths and Difficulties Questionnaire (SDQ). The SDQ assesses mental health problems in childhood and adolescence and consists of 25 items which can be aggregated to four problem subscales. The subscales emotional symptoms and peer relationship problems can be summed up to represent internalized problems, ranging from 0 to 20 points. Values that are classified as borderline or abnormal [36] are integrated to a dichotomous indicator for internalizing problems [37]. The sum of the subscales conduct problems and hyperactivity/ inattention reflects externalizing problems and can be integrated analogously. A total difficulty score can be computed by summing up the four problem subscales, ranging from 0 to 40 points. Higher values indicate greater mental health problems. Validity and reliability of the parent-reported [38] as well as self-reported [39] SDQ in an epidemiological setting in Germany have been both confirmed using the KiGGS study and the BELLA study on mental health and well-being in children and adolescents. Detailed information on the SDQ in the KiGGS study [40] and BELLA study [41] have been previously published.

At follow-up, participants from age 18 completed the five item Mental Health Inventory (MHI-5) which assesses the frequency of five emotions over the past four weeks. The resulting score ranges from 0 to 100 points with a higher score indicating better mental health. This questionnaire has been shown to be equivalent to the full instrument [42]. Additionally, participants from age 18 also completed the nine-item version of the Patient Health Questionnaire (PHQ-9) [43], related to symptoms of major depression occurring in the past two weeks according to the Diagnostic and Statistical Manual of Mental Disorders (DSM-IV, 4th edition [44]). Items rated on a 4-point Likert scale were summed up to a total score ranging from 0 to 27 with a higher score indicating more severe depressive symptoms.

To consider low mental health, for affective mental health problems in childhood as well as young adulthood, two subgroups were built: The subgroup without affective mental health problems comprises participants with values below the cut-off for internalized problems from parent-reported SDQ at baseline [37] and without depressive symptoms according to the PHQ-9 at follow-up (score 0 to 4). Participants whose values for internalized problems at baseline were above the threshold and who indicated moderate to severe depressive symptoms at follow-up (score 10 to 27) were included in the subgroup with affective mental health problems. Participants with either values above the threshold for the SDQ at baseline or the PHQ-9 at follow-up were not considered.

## Measures of cardiovascular health

### CVR factors

SBP, BMI and TC were measured at baseline (age 3 to 17 years) as well as follow-up (age 14 to 28 years) following standardized measurement protocols. Both times, BP measurements were taken twice at a 2-minute interval after 5 min of rest with the participant sitting on a chair, the right forearm placed on the table and the elbow slightly bent, legs uncrossed, and feet placed on the floor. Following a detailed protocol [45], measurements were performed using four different cuff sizes and an automated upper arm oscillometric device (DatascopeAccutorr Plus, Mahwah, NJ), which has been validated in children [46, 47]. The cuff selection rule was updated for examinations at follow-up [48]. At baseline and follow-up, mean SBP was computed from both measurements.

A portable stadiometer (Holtain LTD, United Kingdom) was used to measure height; weight was determined using a calibrated scale (baseline examinations: electronic scale type seca; follow-up examinations: seca mBCA 515/ 514, seca, Hamburg/Germany). BMI was calculated as the ratio of weight in kg to height in cm^2^. TC (in mg/dl) was obtained from venous blood samples by enzymatic determination of serum cholesterol using the CHOD-PAP method.

## Carotid sonography

At follow-up, CIMT and measures of CS were obtained from high-resolution b-mode ultrasound (UF–760AG, Fukuda Denshi Co. Ltd., Tokyo/ Japan) of the common carotid artery with a 5–12 MHz linear array transducer, following current recommendations [49, 50]. After a five-minute resting period, a 10 mm segment 10–15 mm proximal to the bifurcation, was examined in a supine position bilaterally in two planes by a centrally trained physician over at least two heart cycles. The measures of vascular remodelling, i.e. Distensibility coefficient (DC), ß Stiffness Index (ß), Young`s Elastic Modulus (YEM) and Peterson`s Elastic Modulus (Ep) were then calculated using information on CIMT, lumen diameter and BP. While an increase in DC indicates a decrease in stiffness, an increase in Ep, YEM and ß is equivalent to an increase in stiffness. Detailed information on the sonographic measurement technique, assessment of measurement quality, the calculation of measures of CS as well as determinants of successful measurement has been previously published [51]. The investigation of measurement quality also entailed a reliability study including a full set of six examinations who re-examined 15 participants of the KiGGS cohort. Inter- and intra-rater agreement of CIMT measurements and CS parameters was shown to be good or excellent [51].

### Sociodemographic characteristics

Information on citizenship and country of birth of the participant and their parents was considered for migrational background [52]. Three levels of parental education were differentiated, following the simplified International Standard Classification of Education [53]. Because previous research has proposed an association between socioeconomic status (SES) with both, childhood depression [54] as well as CIMT [55, 56], we controlled for SES to rule out confounding. An index for SES was calculated from information of parental education along with parental occupation status and needs-weighted household income [57].

### Statistical analysis

An overview of key indicators related to MH and CVR factors at baseline (age 3 to 17 years) and follow-up (age 14 to 28 years) stratified by sex and age is reported either as means (± standard deviation) or proportions. Previously, centiles for CIMT and CS at follow-up (ages 14 to 28 years) have been modelled (17, 18) using generalized additive models for location, scale and shape, abbreviated as GAMLSS (19). The gamlss package (Version 5.4-3; Stasinopoulos M. and Rigby B., 2022, gamlss.com) in the R software environment (Version 4.1.3; R Foundation for Statistical Computing, Wien, 2022, www.r-project.org), was used to estimate z-scores and the corresponding centiles by age and height for males and females separately. As higher values of all outcomes except for DC equate more unfavorable outcomes, distribution of DC values was inverted to align with the other outcomes.

STATA, Version 17 (StataCorp. 2021. Stata Statistical Software: Release 17. College Station, TX: StataCorp LLC.), was used for the remaining analyses. For the CVR factors, i.e. BMI, SBP and TC, unsmoothed z-scores by sex and age in years were computed. Regression coefficients (ß) from linear regression were used to investigate the longitudinal (11 years from baseline to follow-up) and cross-sectional associations of the measures of MH with CVR factors, CIMT and CS at follow-up, in adolescence and young adulthood. For the cross-sectional analyses, the sample was split into two subsamples: In the younger subsample (aged 14 to 17 years), SDQ was used as a measure of MH in adolescence. Therefore, associations between SDQ and CVR factors were investigated. In the adult subsample (age 18 to 28 years), PHQ-9 and MHI-5 are measures of MH in adulthood. Thus, cross-sectional analyses of the adult subsample investigated the associations between PHQ-9 and MHI-5 and the CVR factors.

As the z-scores from all outcomes were computed in strata of sex and age, the resulting regression coefficients are also adjusted for sex and age. Because all CVR factors have been converted to z-scores (see above), the analyses yield standardized regression coefficients. In a next step, all models were adjusted also by SES and CVR factors at baseline, i.e. BMI, SBP and TC.

In a subsample, we investigated whether participants with affective mental health problems in childhood, i.e. at baseline, as well as young adulthood, i.e. at follow-up, (*n* = 100) were at greater risk for elevated CVR factors or increased CIMT or CS ≥ 90th centile compared to participants without mental health problems at both points in time (*n* = 999). For this analysis, participants with mental health problems at either baseline or follow-up only were excluded. Odds ratios (OR) and 90% confidence intervals (CI) from logistic regressions are reported.

## Results

In Table 1, key characteristics of the sample of 4,361 participants of the KiGGS cohort at baseline (ages 3 to 17 years) and at follow-up (ages 14 to 28 years, on average follow-up after 10.92 years, range: 10.36 to 12.16 years), are presented. Stratified by sex and age groups, measures of mental health, CVR factors, and measures of vascular remodeling as well as socio-demographics are described.

**Table 1:** Characteristics of the study sample of KiGGS-cohort participants

		Total	Female		Male	
	N	4,361	889	1,267	880	1,325
Baseline	**Age at baseline**	**3–17**	**3–6**	**7–17**	**3–6**	**7–17**
	Total cholesterol, mmol/l (SD)	4.30 (0.72)	4.36 (0.71)	4.37 (0.76)	4.28 (0.70)	4.20 (0.68)
	BMI in kg/m², mean (SD)	17.45 (3.30)	15.68 (1.50)	18.66 (3.71)	15.73 (1.37)	18.66 (3.71)
	SBP in mmHg, mean (SD)	104.32 (11.06)	98.27 (7.80)	107.48 (9.98)	98.21 (7.44)	109.22 (12.10)
	DBP in mmHg, mean (SD)	63.25 (7.80)	60.25 (6.79)	65.28 (7.38)	59.66 (6.78)	65.60 (7.92)
	SDQ- Total difficulty score, mean (SD)	7.69 (4.66)	7.26 (4.04)	7.12 (4.62)	8.40 (4.41)	8.06 (5.12)
	SDQ- Internalized problems, mean (SD)	2.88 (2.58)	2.62 (2.31)	2.97 (2.59)	2.85 (2.42)	3.00 (2.82)
	SDQ- Externalized problems, mean (SD)	1.80 (0.40)	1.82 (0.38)	1.85 (0.36)	1.74 (0.44)	1.78 (0.41)
	Migration background (%)	17.10	15.30	17.79	17.02	17.69
	Parental Education^*^: Low (%)	12.38	9.73	16.12	9.22	12.74
	Medium (%)	57.05	59.62	55.20	57.82	56.56
	High (%)	30.57	30.65	28.67	32.96	30.71
Follow-up	**Age at follow-up**	**14–28**	**14–17**	**18–28**	**14–17**	**18–28**
	Total cholesterol, mmol/l (SD)	4.42 (0.91)	4.37 (0.81)	4.84 (0.92)	3.92 (0.71)	4.40 (0.88)
	HDL cholesterol, mmol/l (SD)	1.41 (0.31)	1.44 (0.26)	1.61 (0.34)	1.26 (0.23)	1.29 (0.25)
	BMI in kg/m², mean (SD)	22.93 (4.23)	21.69 (3.43)	23.36 (4.62)	21.78 (3.97)	24.17 (4.15)
	SBP in mmHg, mean (SD)	120.30 (10.97)	113.83 (8.21)	118.14 (9.87)	119.28 (10.11)	127.59 (10.24)
	DBP in mmHg, mean (SD)	70.97 (7.16)	68.74 (6.45)	71.29 (7.17)	69.32 (6.83)	73.35 (7.08)
	Current smoking (%)	24.14	10.87	29.16	12.71	36.68
	SDQ- Total difficulty score, mean (SD)	-	6.34 (4.69)	-	6.98 (5.00)	-
	SDQ- Internalized problems, mean (SD)	-	3.17 (2.96)	-	2.68 (2.82)	-
	SDQ- Externalized problems, mean (SD)	-	1.92 (0.27)	-	1.85 (0.36)	-
	MHI-5 score, mean (SD)	-	-	72.18 (15.52)	-	76.85 (13.66)
	PHQ-9 score, mean (SD)	-	-	6.17 (4.28)	-	4.89 (3.70)
	Persistent MH problems (%)	-	-	11.76	-	7.10
	CIMT in mm, mean (SD)	0.54 (0.05)	0.52 (0.04)	0.53 (0.05)	0.54 (0.05)	0.55 (0.05)
	DC in 10^− 3^ kPa, mean (SD)	48.08 (12.16)	56.02 (12.35)	48.50 (11.11)	49.69 (10.88)	41.02 (9.70)

SD: standard deviation. SBP: systolic blood pressure; DBP: diastolic blood pressure; SDQ: Strength and Difficulties Questionnaire; CIMT: carotid intima-media thickness; DC: distensibility coefficient; HDL: High-density lipoprotein; BMI: body mass index. MH: Mental health

Table 2 gives an overview of the longitudinal (11 years from baseline to follow-up) and cross-sectional associations of the measures of mental health with CVR factors, i.e. BMI, SBP and TC, and biomarkers for vascular remodeling, i.e. CIMT and DC, at age 14 to 28 years. Our analyses yielded significant, but very small longitudinal associations (-0.03≥ß≤ 0.03) between the childhood SDQ total difficulty score, as well as the internalizing and externalizing subscales at ages 3 to 17 years with CVR factors, i.e. SBP, BMI and TC, in adolescence and young adulthood (ages 14 to 28 years). Cross-sectionally, somewhat stronger associations of up to -0.15 for a one-point increase in externalizing problems subscale (ranging from 0 to 20) on the TC z-score could be observed in the younger subsample aged 14 to 17 at follow-up. Among the adult subsample aged 18 years and older at follow-up, overall mental health, measured by the MHI-5, was not associated with SBP, BMI or TC. Depressive symptoms, measured by the PHQ-9, were also not associated cross-sectionally with TC. However, depressive symptoms at follow-up were significantly associated with SBP: A one-point increase in the summary score of the PHQ-9 (ranging from 0 to 27) was significantly associated with a z-score decrease of 0.26. The strength of this association was considerably reduced (ß= -0.03) when adjusting for SES and CVR factors at baseline.

**Table 2:** Associations of mental health measures in adolescence and young adulthood with cardiovascular risk factors and with measures of vascular remodeling

Measures of mental health				Outcomes at follow-up Regression coefficients				
				TC	SBP	BMI	CIMT	DC
Longitudinal: MH at baseline	Adjusted: age, sex	SDQ	Total difficulty score	-0.01*	0.00	0.02*	0.00	0.00
			Internalizing problems	0.00	0.01	0.03*	0.00	0.00
			Externalizing problems	-0.03*	0.00	0.02*	0.00	0.00
	Further adjusted ^1^	SDQ	Total difficulty score	0.00	0.01*	0.01*	0.00	0.00
			Internalizing problems	0.00	0.00	0.01	0.00	0.00
			Externalizing problems	-0.01*	0.02*	0.02*	0.01	-0.01
Cross-sectional: MH at follow-up	Adjusted: age, sex	SDQ	Total difficulty score	0.00	-0.01	0.01*	0.00	0.00
			Internalizing problems	0.02*	-0.02*	0.01	0.00	0.00
			Externalizing problems	-0.15*	0.00	0.02*	0.00	0.00
			PHQ-9	0.00	-0.26*	0.01*	0.00	0.00
			MHI-5	0.00*	0.00*	0.00	0.00	0.00
	Further adjusted ^1^	SDQ	Total difficulty score	0.00	-0.01*	0.01*	0.00	0.00
			Internalizing problems	0.02*	-0.02*	0.01	0.00	0.00
			Externalizing problems	-0.02*	-0.01	0.02*	0.01	0.00
			PHQ-9	0.00	-0.03*	0.01*	0.00	-0.01
			MHI-5	0.00*	0.00*	0.00	0.00	0.00

SES: socio-economic status. SDQ: Strength and Difficulties Questionnaire, parent-reported. PHQ: Patient Health Questionnaire. MHI: Mental Health Inventory. SBP: Systolic blood pressure. BMI: Body Mass Index. TC: Total Cholesterol. CIMT: carotid intima-media thickness. DC: distensibility coefficient (inverted so that higher values imply adverse functional changes). ^**1**^ Adjusted for age, sex, SES and z-scores of SBP, BMI and TC at baseline (cross-sectional) or follow-up (longitudinal). * *p* < 0.05. Baseline mental health (MH) measures *N* = 4,361, Follow-up SDQ *N* = 1,799, PHQ-9 and MHI-5 *N* = 2,457

Longitudinally, no measure of the SDQ at ages 3 to 17 years was associated with CIMT or DC one decade later, also when further adjusting for SES and CVR at baseline. Similar results were obtained for cross-sectional associations of SDQ with CIMT and DC when investigating the younger subsample aged 14 to 17 years at follow-up. Cross-sectional analyses of the subsample aged 18 to 28 years at follow-up showed no association between overall mental health, measured by the MHI-5, or depressive symptoms, measured by the PHQ-9, with CIMT or DC. Analyses of three other parameters of CS, i.e. ß, YEM and Ep, revealed similar associations as DC (see Supplemental Table 1).

**Fig. 1:**
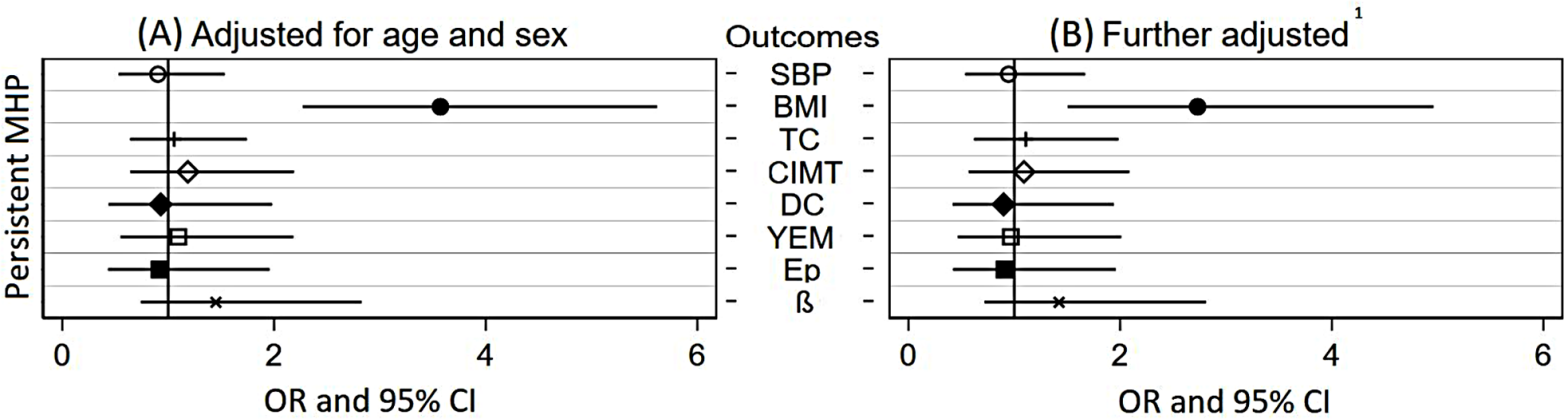
Association of affective mental health problems in childhood as well as young adulthood with cardiovascular risk factors and measures of vascular remodeling. Note: Odds ratios (OR) and 95% confidence intervals (CI) from logistic regressions for affective mental health problems in childhood as well as young adulthood and elevated cardiovascular outcomes ≥ 90th centile, i.e. systolic blood pressure (SBP), Body Mass Index (BMI), Total Cholesterol (TC), Carotid Intima-Media Thickness (CIMT), Distensibility Coefficient (DC), Young`s Elastic Modulus (YEM), Peterson`s Elastic Modulus (Ep) as well as ß Stiffness Index (ß). Comparison group: no mental health problems at baseline or follow-up. ^1^Further adjusted for baseline SBP, BMI, TC and socio-economic status

In a subgroup analysis, we also examined whether participants with affective mental health problems in childhood as well as young adulthood were at greater risk for adverse outcomes than their peers without mental health problems. We did not find increased odds for elevated TC, SBP, CIMT or CS in participants with persistent mental health problems (see Fig. 1). Even so, the OR for elevated BMI was significantly increased, also when controlling for SES and baseline CVR factors (OR 2.46, 95% CI 1.31–4.62).

## Discussion

This study investigated the association between different aspects of mental health with CVR factors as well as biomarkers of vascular remodeling in a population-based sample of adolescents and young adults. We found a pattern of associations between childhood as well as adolescent mental health and CVR factors in adolescence and young adulthood, i.e. SBP, BMI and TC. However, in this general population sample we found no evidence for an association between mental health and vascular remodeling in the young.

Similar to the results presented in this study, previous studies have also found mental health in the young to be associated to CVR factors but not vascular remodelling. A cross-sectional study reported no evidence for an association between mental health and CIMT as well as PWV, but a link between mental health and obesity [31]. This is in line with results from a large cohort study suggesting that childhood depression and obesity are linked through lifestyle behaviours and sociodemographic characteristics [58]. For example, mental health symptoms may change eating and physical activity patterns, thereby increasing the risk of obesity and hyperlipidaemia. Another cross-sectional study found symptoms of depression and anxiety to be independent of CIMT as well as measures of CS, but depression associated with HDL cholesterol as well as triglycerides [32]. Previously, depressive symptoms have also been linked to increased childhood BP [33]. Our study confirms these associations: We found small associations between childhood mental health and CVR factors one decade later, i.e. SBP, BMI, and TC. We found depressive symptoms to be significantly associated with lower SBP in young adults in the KiGGS cohort. This association seems counterintuitive at first. However, a recent study of more than 500,000 participants of the UK biobank also found higher SBP related to better mood ratings [59]. Congruent with their results from functional magnetic resonance imaging and building on previous research [60, 61], the authors propose that increased BP might be associated with weakened processing of emotional valence.

Whereas previous studies by and large align in their findings regarding the association between mental health and CVR in the young, the same does not hold true for the link between mental health and vascular remodelling. In addition to the studies mentioned above, several other studies also did not find childhood overall mental health or depressive symptoms specifically to be associated with CIMT [31, 32, 35], carotid distensibility [32] or PWV [31, 33, 34] later on. Then again, a recent study in a large population-based cohort found that emotional symptoms in adolescence were associated with a CIMT increase of 2 to 3 μm at age 18 [28]. As in our study, the SDQ was used to assess childhood mental health problems. Results from the Cardiovascular Risk In Young Finns Study also reported sex differences, finding an association between adolescent depressive symptoms and CIMT in young adulthood only in males [27]. However, when later considering depressive symptoms from several points in time and using more recent CIMT measurements of the same cohort, this association could not be confirmed [30].

While evidence in the young is inconclusive, numerous studies have reported an association between depressive symptoms and subclinical atherosclerosis in older individuals [11, 13–16]. As vascular alterations gradually accumulate over a long period of time, the association between depressive symptoms and vascular remodeling might manifest only after decades. This is supported by a recent meta-analysis of 19 studies with more than 32,000 participants which concluded that depressive symptoms are linked to CIMT consistently from age 50 on [62]. As participants of the KiGGS cohort were between 14 and 28 years old when undergoing carotid sonography, they might not have been old enough to have developed vascular alterations in response to low mental health. There are several other reasons why our study might not have detected an association between mental health and vascular remodelling. In this study only carotid IMT data are available and not aortic measurements. It has been hypothesized that structural changes occur in the aorta earlier than in the carotid arteries [63]. Results from the Muscatine Offspring Study propose the use of aortic and carotid intima-media thickness as complementary [64]. Furthermore, the KIGGS cohort included healthy individuals who were asked about their current mental health. The number of participants with moderate to severe depressive symptoms was small and no information with regard to diagnoses of clinical depression or history of depression was obtained. The strength of the association between mental health and adverse vascular changes has been shown to be dependent on the severity of depressive symptoms as well as their duration. For example, one large population-based study suggests that history of depression might be more relevant for vascular changes than current depressive symptoms [65]. Also, one small study in young women found that recurrent depressive episodes before the age of 30 were associated with higher CIMT [66].

Even though this study did not find a direct link between mental health and vascular remodelling in the young, the results emphasize the importance of maintaining good physical as well as mental health in order to preserve ideal CV health. While behavioural risk factors as well as physical health, such as smoking, obesity and physical activity, are widely recognized for their relevance concerning preventive efforts, mental health is given much less attention, especially amongst the young. Considering a longer time frame, an analysis of 70,000 primary care patients revealed that individuals with depression had a higher 10- and 30-year absolute CVR compared to individuals with controlled depression or without depression [67]. This evidence in mind, it is not surprising that depression was graded a prognostic factor for acute coronary syndromes in a position paper of the European Society of Cardiology [68].

### Strengths and limitations

This study utilized data on carotid function and structure in unselected young adults and adolescents from a large national health examination survey. High validity as well as completeness [51] were achieved by conducting carotid sonography in adherence to recommendations [49, 50]. This is one of few large, population-based studies with longitudinal data on mental health from childhood to adolescence and young adulthood and CVR factors as well as subclinical carotid markers of atherosclerosis. To assess mental health status, age-appropriate instruments were used: Parent-reported SDQ has been used previously when investigating the association of childhood mental health problems and vascular remodeling [28, 31]. It is a widely used behavioral screening questionnaire to assess mental health problems in children and adolescents (45). For adults, the MHI-5 and PHQ-9 were used. While the former has been shown to be a reliable measure of general mental health [69], the latter provides information about depressive symptoms specifically, being used in studies similar to ours [14]. The MHI-5 has also been shown to be equivalent to the full instrument [42] and is used as a screener for mood and anxiety disorders [70].

Several limitations must be mentioned. First, the shift in measurement instrument from childhood to young adulthood is undesirable, but unavoidable in order to consider age-specific symptoms and questionnaires. Carotid plaques were not investigated as their occurrence is exceedingly rare among young individuals and very unlikely without any adverse carotid remodeling as examined [71]. As CIMT was measured at follow-up, but not at baseline, CIMT progression could not be studied. Genetic dispositions towards CVD as well as mental health could not be examined as no genetic testing was performed in KiGGS. What is more, differences in ethnicity were not investigated because the KiGGS cohort is composed almost entirely of Caucasians. Finally, this study draws on longitudinal data from an observational study. Thus, we examined whether associations could be observed between predictor variables and outcomes. Randomized controlled trials are necessary to make statements on causality.

## Conclusion

This study found no association between mental health or depressive symptoms and CIMT as well as CS in a population-based sample of adolescents and young adults. However, we found associations between childhood mental health and CVR factors one decade later. Thus, low mental health may predispose individuals for higher cardiovascular risk profiles already in adolescence and young adulthood. This underlines the importance of behavioural prevention strategies which should focus on both mental and physical health of the young before atherosclerotic damage may manifest later in life.

## Electronic supplementary material

Below is the link to the electronic supplementary material.

Supplementary Material 1
