# Supplementary material for: Association of mental health in childhood, adolescence and young adulthood with cardiovascular risk factors and carotid remodeling below age 30 - results from the KiGGS cohort study
Source: Eur J Epidemiol. 2025 Jan 3;40(1):17–26. doi: 10.1007/s10654-024-01189-3 (PMC11799030; doi:10.1007/s10654-024-01189-3)
Supplement: Supplementary file 1 — Supplementary Material 1 [file 10654_2024_1189_MOESM1_ESM.docx]

Supplemental table 1. Longitudinal and cross-sectional associations of z-scores of MH measures in adolescence and young adulthood with additional carotid stiffness parameter from linear regression models.

|  |  |  |  | **Regression coefficients for**  **measures at follow-up** | | |
| --- | --- | --- | --- | --- | --- | --- |
|  |  |  | **Measures of MH** | **ß** | **YEM** | **Ep** |
| **Longitudinal:**  **MH at Baseline** | **Adjusted: age, sex** | SDQ | Total difficulty score | 0.00 | 0.00 | 0.00 |
|  |  |  | Internalizing problems | 0.00 | 0.00 | 0.00 |
|  |  |  | Externalizing problems | 0.00 | 0.00 | 0.00 |
|  | **Further adjusted ^1^** | SDQ | Total difficulty score | 0.00 | 0.00 | 0.00 |
|  |  |  | Internalizing problems | 0.00 | 0.00 | 0.00 |
|  |  |  | Externalizing problems | 0.00 | 0.00 | 0.01 |
| **Cross-sectional:**  **MH at Follow-up** | **Adjusted:**  **age, sex** | SDQ | Total difficulty score | 0.00 | 0.00 | 0.00 |
|  |  |  | Internalizing problems | -0.01 | 0.00 | 0.00 |
|  |  |  | Externalizing problems | 0.00 | 0.00 | 0.00 |
|  |  |  | PHQ-9 | 0.01 | 0.00 | 0.00 |
|  |  |  | MHI-5 | 0.00 | 0.00 | 0.00 |
|  | **Further adjusted ^1^** | SDQ | Total difficulty score | 0.00 | 0.00 | 0.00 |
|  |  |  | Internalizing problems | -0.01 | 0.00 | 0.00 |
|  |  |  | Externalizing problems | 0.00 | 0.00 | 0.00 |
|  |  |  | PHQ-9 | 0.01 | 0.01 | 0.01 |
|  |  |  | MHI-5 | 0.00 | 0.00 | 0.00 |

SES: socio-economic status. SDQ: Strength and Difficulties Questionnaire, parent-reported. PHQ: Patient Health Questionnaire. MHI: Mental Health Inventory. SBP: Systolic blood pressure. BMI: body mass index. TC Total Cholesterol. ß: Beta Stiffness Index. YEM: Young’s Elastic Modulus Ep: Peterson’s Elastic Modulus. ^1^ Adjusted for age, sex, SES and z-scores of SBP, BMI and TC at baseline (cross-sectional) or follow-up (longitudinal). * p< 0.05. Baseline mental health (MH) measures N=3,991, Follow-up SDQ N=1,633, PHQ-9 and MHI-5 N=2,261.
